# Comparative Proteomic Analysis Reveals Varying Impact on Immune Responses in Phorbol 12-Myristate-13-Acetate-Mediated THP-1 Monocyte-to-Macrophage Differentiation

**DOI:** 10.3389/fimmu.2021.679458

**Published:** 2021-06-21

**Authors:** Sneha M. Pinto, Hera Kim, Yashwanth Subbannayya, Miriam S. Giambelluca, Korbinian Bösl, Liv Ryan, Animesh Sharma, Richard K. Kandasamy

**Affiliations:** ^1^ Centre of Molecular Inflammation Research (CEMIR), and Department of Clinical and Molecular Medicine (IKOM), Norwegian University of Science and Technology, Trondheim, Norway; ^2^ Center for Systems Biology and Molecular Medicine, Yenepoya (Deemed to be University), Mangalore, India; ^3^ Department of Infectious Diseases, Medical Clinic, St. Olavs Hospital, Trondheim, Norway; ^4^ Proteomics and Modomics Experimental Core, PROMEC, Norwegian University of Science and Technology and the Central Norway Regional Health Authority, Stjørdal, Norway

**Keywords:** monocyte, macrophage, TLR signaling, innate immune signaling, functional networks, pathways, differentiation

## Abstract

Macrophages are sentinels of the innate immune system, and the human monocytic cell line THP-1 is one of the widely used *in vitro* models to study inflammatory processes and immune responses. Several monocyte-to-macrophage differentiation protocols exist, with phorbol 12-myristate-13-acetate (PMA) being the most commonly used and accepted method. However, the concentrations and duration of PMA treatment vary widely in the published literature and could affect the probed phenotype, however their effect on protein expression is not fully deciphered. In this study, we employed a dimethyl labeling-based quantitative proteomics approach to determine the changes in the protein repertoire of macrophage-like cells differentiated from THP-1 monocytes by three commonly used PMA-based differentiation protocols. Employing an integrated network analysis, we show that variations in PMA concentration and duration of rest post-stimulation result in downstream differences in the protein expression and cellular signaling processes. We demonstrate that these differences result in altered inflammatory responses, including variation in the expression of cytokines upon stimulation with various Toll-like receptor (TLR) agonists. Together, these findings provide a valuable resource that significantly expands the knowledge of protein expression dynamics with one of the most common *in vitro* models for macrophages, which in turn has a profound impact on the immune as well as inflammatory responses being studied.

## Introduction

Macrophages and their precursors- monocytes, mediate innate immune responses and inflammatory processes and contribute to adaptive immunity through antigen presentation (1, 2). Monocytes in the blood circulation migrate to the site of infection/inflammation and differentiate into macrophages for effective host defense, tissue remodeling, and repair (3). Furthermore, macrophages exhibit a high level of plasticity, depending on their local microenvironment, specialized functions, and varied phenotype acquired (1, 4).

Several models are employed to study the mechanisms of immune modulation in monocytes and macrophages. The most frequently used include primary peripheral blood mononuclear cells (PBMCs) and monocyte cell lines. However, due to donor-to-donor variations and technical disparities involved in the handling of PBMCs *in vitro*, the human leukemia monocytic cell line, THP-1, is widely accepted and used as a monocyte/macrophage model. Previous studies have demonstrated that THP-1 cells can be differentiated into macrophage-like cells using phorbol-12-myristate-13- acetate (PMA), resulting in a phenotype that markedly resembles PBMC monocyte-derived macrophages (MDMs) (5, 6). These cells have been shown to have comparable cytokine production, metabolic and morphological properties, including differential expression of macrophage surface markers such as *CD14*, CD11b (*ITGAM*), and scavenger receptors- *CD163*, *MSR1*, and *SCARB2* (5, 7–10). Nonetheless, depending on the parameters of the differentiation protocol employed, such as the concentration (ranging from 5 to 400 ng/mL) and duration of incubation (1 to 5 days) with PMA; the degree of differentiation and the functional changes may vary significantly (5, 11–15).

At the molecular level, multiple proteins, including growth factors, antigenic markers, chemokine-receptors, cytokines, and cell adhesion molecules, are known to govern and reflect underlying monocyte-macrophage differentiation processes (16–20). However, the effect of various differentiation protocols on the cellular proteome and intracellular signaling networks during monocyte-to-macrophage differentiation remains poorly understood. Hence, it is crucial to determine the most suitable differentiation conditions when using these cells as a model system, as this can significantly impact their response to various innate immune stimuli.

Quantitative high-resolution mass spectrometry-based proteomic approaches have been widely employed to investigate the proteomes of monocytes and macrophages as well as altered cellular proteomes and complex cellular/biological mechanisms in several biological conditions (21–23). However, to date, no studies have directly compared the differences in protein expression dynamics with respect to concentration, treatment time and duration of incubation in various PMA-mediated differentiation protocols.

In the present study, we evaluate the effect of three PMA-based differentiation protocols on the changes in the proteome profiles upon THP-1 differentiation using stable isotope dimethyl labeling quantitative proteomics. We observed alterations in the proteome expression profile that in turn significantly impacted cellular processes such as cell migration, regulation of immune responses, and metabolic processes. Overall, these cellular differences had an effect on the extent of innate immune and inflammatory responses. We demonstrate that various differentiation conditions, such as concentration and incubation time, prior to any stimuli, are critical consideration factors responsible for heterogeneity of the cell culture.

## Experimental Procedures

### Cell Culture and Differentiation

Human THP-1 monocytic cells (ATCC) were cultured in RPMI 1640 (Sigma-Aldrich) medium containing 10% heat-activated fetal calf serum (FCS), 2 mM L-glutamine, 100 nM penicillin/streptomycin (Thermo Fisher Scientific) and 50 µM β-mercaptoethanol (Sigma-Aldrich). The cells were maintained in a humidified 37°C, 5% CO_2_ incubator. THP-1 cells were counted using Z2 Coulter particle count and size analyzer (Beckman Coulter) and seeded at a density of 0.2 x 10^6^ cells/ml. THP-1 cells were differentiated into resting macrophages by resuspending the cells in growth medium containing 5 or 50 ng/ml phorbol-12-myristate-13-acetate (PMA; Sigma-Aldrich) and cultured for indicated time periods. The treatment conditions included the following- Condition A: 50 ng/ml PMA for 72 hours followed by 48 hours rest (5 days); Condition B: 50 ng/ml PMA overnight (16 hours) followed by 48 hours rest (Condition B); and Condition C: 5 ng/ml PMA for 48 hours followed by 3 hours rest. The process of differentiation was enhanced by removing the PMA-containing media and adding fresh, complete RPMI 1640 media to the cells. Treatment with 50 ng/ml PMA for 72h followed by 2 days rest in media without PMA has been well described (24–27). It has also been shown that the same concentration treated for a shorter duration has a similar effect, and previous reports suggest lower concentrations may be sufficient to induce differentiation while minimizing off-target effects. The rationale for choosing condition C is based on an earlier study that reported treatment of THP-1 cells with 5 ng/ml PMA for 48 h was sufficient to induce stable differentiation without undesirable gene upregulation upon secondary stimuli (12).

### Phase-Contrast Microscopy

THP-1 cells were seeded at a density of 0.2 x 10^6^ cells/ml in cell culture Cellvis glass-bottom plates and treated with PMA and rested at indicated concentrations and duration. The morphological characteristics of undifferentiated and differentiated THP-1 cells were captured by EVOS FL Auto Cell Imaging System 2 (Thermo Fisher Scientific) using a 40x objective lens and were processed by ImageJ software (W.S. Rasband, National Institutes of Health, Bethesda, MD).

### Flow Cytometry

THP-1 cells were plated in 12-well culture plates (Corning Costar) as described above. The cells were washed three times with 1X PBS and detached from plates using Accutase (A6964; Sigma-Aldrich) incubation for 15 minutes at 37°C. The detached cells were collected on ice and then centrifuged. Human TruSatin FcX™, FcR Blocking Reagent (1 µg IgG/10^6^ cells in 100 µl staining volume; BioLegend, #422301) was applied to decrease the non-specific binding for 10 minutes on ice. Cells were subsequently stained with Brilliant Violet 785™ anti-human CD14 Antibody (1:1000; BioLegend, #301840), APC anti-human CD86 Antibody (1:1000; BioLegend, #305412), and PE anti-human CD11b Antibody (1:1000; BioLegend, #301306) for 30 minutes in the dark. The cells were then fixed and permeabilized with 1% paraformaldehyde (PFA). Flow cytometry data were acquired on a BD LSRII flow cytometer (BD Biosciences) with FACS Diva software (BD) and analyzed using FlowJo software (FlowJo, LLC).

### Cell Lysis and Sample Preparation for Mass Spectrometry

The cells for proteomic analysis were cultured, as described above. After the indicated time points of PMA incubation followed by resting, the media was discarded, and the cells were washed thrice with ice-cold PBS. The cells were lysed and harvested using SDS lysis buffer (2% SDS, 50 mM TEABC) and sonicated using a probe sonicator (Branson Digital Sonifier) on ice for 5-10 minutes (20% amplitude, 10 cycles). The lysates were heated at 95°C for 10 minutes, allowed to cool to room temperature, and centrifuged at 12,000 rpm for 10 minutes. The protein concentration in the lysates was estimated by the Bicinchoninic acid (BCA) assay (Pierce, Waltham, MA). 70 µg proteins from each condition were reduced and alkylated with 10 mM dithiothreitol (DTT) at 60°C for 20 minutes and 20 mM iodoacetamide (IAA) at room temperature for 10 minutes in the dark, respectively. The protein samples were then subjected to acetone precipitation with five volumes of chilled acetone at -20°C for 6 hours. Protein pellets were obtained by centrifugation at 12,000 rpm for 15 minutes at 4°C, resuspended in 100mM TEABC and subjected to trypsin digestion with sequencing grade trypsin (1:20) (Sigma Aldrich) overnight at 37°C. Digestion efficiency was checked using SDS-PAGE analysis of pre- and post digest samples (10 µg equivalent loaded on gel).

### Dimethyl-Labeling of Tryptic Peptides

20 µg tryptic peptides obtained from Condition A, B, and C were subjected to reductive dimethylation with 4% (vol/vol) formaldehyde (CH_2_O) (Light), (CD_2_O) (Medium) or (^13^CD_2_O) (Heavy) labels, respectively (28). Following this, 4 µl of 0.6 M sodium cyanoborohydride (NaBH_3_CN) was added to the samples to be labeled with light and intermediate labels, and 4 µl of 0.6 M sodium cyanoborodeuteride (NaBD_3_CN) to the sample to be heavy labeled respectively. The mixture was incubated for 1 hour at room temperature. The labeling efficiency was checked by pooling an aliquot of each condition (2 µg) and analyzing on mass spectrometer. Post label check analysis, the reaction was quenched with 16 µl of 1% ammonia. Finally, 8 µl formic acid was added. The three differentially labeled samples were pooled and desalted using C_18_ StageTip, evaporated to dryness under vacuum, acidified using 2% TFA and subjected to StageTip based Strong-cation exchange (SCX) fractionation with four plugs of SCX material as described previously (29).

### Mass Spectrometry Analysis

Mass spectrometric analyses of the SCX fractions were carried out using a Q Exactive HF Hybrid Quadrupole-Orbitrap mass spectrometer (Thermo Fisher Scientific, Bremen, Germany) coupled to Easy-nLC1200 nano-flow UHPLC (Thermo Scientific, Odense, Denmark). The data were acquired for each of the samples in biological quadruplicates. Briefly, tryptic peptides obtained from StageTip-based SCX fractionation were reconstituted in 0.1% formic acid and loaded on an Acclaim PepMap 100 2 cm (3 µm C18 Aq) trap column (Thermo Fisher Scientific). Peptide separation was carried out using Acclaim PepMap 100 C18 HPLC Column, 50 cm (Thermo Fisher Scientific) heated to 40°C using an integrated column oven. Peptide separation was carried out at a flow rate of 250 nl/min using a binary solvent system containing solvent A: 0.1% formic acid and solvent B: 0.1% formic acid, 80% acetonitrile. A linear gradient of 5-30% solvent B over 150 minutes, followed by a linear gradient of 30-95% solvent B for 5 minutes, was employed to resolve the peptides. The column was re-equilibrated to 5% solvent B for an additional 20 minutes. The total run time was 180 minutes. Data were acquired in positive mode using a data-dependent acquisition method wherein MS1 survey scans were carried out in 350-1650 m/z range in Orbitrap mass analyzer at a mass resolution of 120,000 at m/z 200. The peptide charge state was set to 2-6, and dynamic exclusion was set to 30 s along with an exclusion width of ± 20 ppm. MS/MS fragmentation was carried out for the most intense precursor ions selected at top speed data-dependent mode with a maximum cycle time of 3 seconds. HCD fragmentation mode was employed with a collision energy of 30% and detected at a mass resolution 15,000 at m/z 200. Internal calibration was carried out using a lock mass option (m/z 445.1200025) from ambient air (30).

### Data Analysis

Protein identification and quantification were performed using Proteome Discoverer Version 2.3 with the following parameters: carbamidomethyl of cysteine as a fixed modification, and oxidation of methionine, deamidation of asparagine and glutamine, acetylation (protein N terminus), quantitation labels Dimethyl, Dimethyl:2H4 and Dimethyl:2H(6)13C(2) on N-terminal and/or lysine were set as variable modifications. Trypsin was specified as a proteolytic enzyme with a maximum of 2 missed cleavages allowed. The searches were conducted using the SequestHT node against the Uniprot-Trembl Human database (v2017-10-25, 42,275 entries), including common contaminants (245 entries). Mass accuracy was set to 10 ppm for precursor ions and 0.02 Da for MS/MS data. For FDR calculation, the MS data were searched against the decoy database. Identifications were filtered at a 1% false-discovery rate (FDR) at the peptide level, accepting a minimum peptide length of 7 amino acids. Quantification of identified proteins referred to the razor and unique peptides and required a minimum ratio count of 2. Dimethyl-based relative ratios were extracted for each protein/condition using the Minora Feature Detector node and were used for downstream analyses.

### Bioinformatics Analysis

Protein abundances across multiple replicates were scaled, log-transformed, normalized using the cyclic loess method, and analyzed for differential expression in limma v3.38.3 (25) in R/Bioconductor (v3.5.2, https://www.r-project.org/; v3.8 https://bioconductor.org). The treatment conditions were used for generating contrasts. Proteins expressed with a log2-fold change ≥ ± 2 were considered as differentially expressed. Volcano plots were drawn using the EnhancedVolcano R package (v 1.0.1), and proteins with –log10 (p-value) ≥ 1.25 were considered to be significant. Heatmaps of expression data and *k*-means clustering was carried out in Morpheus using Euclidean complete linkage (https://software.broadinstitute.org/morpheus/). Significant clusters of genes that were overexpressed in conditions A-C were extracted, and enriched biological processes were identified using Enrichr (https://amp.pharm.mssm.edu/Enrichr/). Hypergeometric enrichment-based pathway analysis was carried out using ReactomePA (1.28.0) in R (v3.6.0, https://www.r-project.org/)/Bioconductor (v3.9 https://bioconductor.org) with clusterProfiler 3.12.0 (26). Genesets with a minimum of 15 genes were considered for the analysis. The plot was visualized using the ggplot2 package (v3.2.1) (https://cran.r-project.org/web/packages/ggplot2). Significantly changing clusters for each condition from the *k*-means clustering analysis were subjected to network analysis using STRING in Cytoscape (version 3.7.1). The network properties were calculated using NetworkAnalyzer and visualized in Cytoscape using betweenness centrality and degree parameters. The entire networks were further subjected to clustering to identify significant sub-clusters using the MCODE app (v1.5.1) (27) in Cytoscape. The parameters used for clustering included degree cutoff of 2, node score cutoff of 0.2, *k*-core of 2, and Max. Depth of 100. Kinome trees were drawn using KinMap (28) (http://kinhub.org/kinmap). Gene lists for functions such as phagocytosis, reactive oxygen species, and inflammasome complex were obtained from the Molecular Signatures Database (MSigDB, v7.0, https://www.gsea-msigdb.org/gsea/msigdb) (31). Protein kinase and phosphatase lists were obtained, as described previously (32).

### Inflammasome Activation Assay

THP-1 cells were differentiated into macrophage-like phenotype with the three PMA differentiation protocols described above in biological triplicates. PMA- treated cells were primed with LPS (100 ng/ml) for 2 hours and stimulated with Nigericin (5 μg/ml) for a further 2 hours. Cell pellets and supernatants were harvested for IL1β or LDH release and analyzed by western blot or LDH cytotoxicity detection kit (MK401 Takara) according to the manufacturer’s instructions. For western blot analysis, supernatant was collected and stored at -80 °C until further analysis. Cell lysates were prepared in RIPA lysis buffer (150 mM NaCl, 50 mM Tris-HCl, pH 7.5, 1% Triton X-100, 5 mM EDTA, protease inhibitors, and phosphatase inhibitors) and the protein concentrations were determined by BCA assay (Pierce, Waltham, MA).

### Western Blot Analysis

Protein samples were run on pre-cast NuPAGE™ Bis-Tris gels (Invitrogen) with 1 x MOPS buffer (Invitrogen) and transferred on nitrocellulose membranes, using iBlot^®^2 Gel Transfer Device (Invitrogen). Membranes were washed in Tris Buffered Saline with 0.1% Tween-X100 (TBS-T) and blocked with TBS-T containing 5% bovine serum albumin (BSA, Sigma-Aldrich). Membranes were incubated with primary antibodies at 4°C overnight. The following primary antibodies were used: GAPDH (1:5000; ab8245; Abcam), anti-β-Actin (1:5000; cat#6276; Abcam) anti-IRF3 (1:1000; D83B9; cat#4302S; Abcam), anti-TBK1 (1:1000; cat#3504; Cell Signaling Technology), anti- IL1B (1:1000; cat#12242; Cell Signaling Technology) and SQSTM1 (1:1000; cat#PM045; MBL). Membranes were washed in TBS-T and incubated with secondary antibodies (HRP-conjugated, DAKO) for 1 hour at room temperature in TBS-T containing 1% milk or BSA. The blots were developed with SuperSignal West Femto Substrate (Thermo Scientific) and captured with LI-COR Odyssey system (LI-COR Biosciences, Lincoln, NE, USA).

### RNA Isolation and Quantitative Real-Time PCR (qPCR) Analysis

Undifferentiated THP-1 and PMA-differentiated cells (2 x 10^5^ cells) in biological triplicates per condition were stimulated with TLR agonists- CL075 (TLR8; tlrl-c75; Invivogen, 5 µg/ml), CpG2006 (TLR9; tlrl-2006; Invivogen, 10 µM), Flagellin (TLR5; tlrl-stfla; Invivogen, 100 ng/ml), FSL1 (TLR2/6; tlrl-fsl; Invivogen, 100 ng/ml), LPS 0111:B4 (TLR4; tlrl-3pelps; Invivogen, 200 ng/ml), LPS K12 (TLR4; tlrl-eklps; Invivogen, 200 ng/ml), Pam3CSK4 (TLR1/2; tlrl-pms; Invivogen, 200 ng/ml), Poly (I:C) (TLR3; vac-pic; Invivogen, 10 µg/ml), R837 (TLR7; tlrl-imqs; Invivogen, 10 µg/ml), and R848 (TLR7/8; tlrl-r848; Invivogen, 100 ng/ml) for 0, 2 and 4 hours. Post stimulation, total RNA was isolated using RNeasy Mini columns, followed by DNAse digestion (Qiagen), according to the manufacturer’s protocol. The purity and concentrations of RNA were determined using NanoDrop 1000 (Thermo Scientific). cDNA was prepared with High-Capacity RNA-to-cDNA™ (Applied Biosystems). Quantitative real-time PCR (qPCR) analysis was performed on StepOne Plus Real-Time PCR cycler (Thermo Fisher Scientific) using PerfeCTa qPCR FastMix UNG (Quantabio) and FAM Taqman Gene Expression Assays: *IL6* Hs00985639_m1, *IL1B* Hs00174097_m1, *TNF* Hs01113624_g1, *TBP* Hs00427620_m1, *IL8* Hs00174103_m1 (Life Technologies) in 96-well format in technical duplicates. Relative expression compared to the unstimulated control samples and TBP as a housekeeping gene was calculated in R 3.3.2, as described previously (33).

### Multiplexed Cytokine Profiling

Undifferentiated THP-1 and PMA-differentiated cells (2 x 10^5^ cells) in biological triplicates per condition were stimulated with TLR agonists described above for 8 hours. Post stimulation, the supernatants were harvested. Supernatants were diluted 1:20 and analyzed according to the manufacturer’s protocol using the Bio-plex Pro Human Screening Panel 4 plex (IL1β, IL6, IL8, and TNFα) on a Bio-Plex 200 instrument (Bio-Rad Laboratories). Results are presented as means and SD for three independent experiments.

### Statistical Analysis

GraphPad Prism 8.0 was used to perform all statistical analyses and determine p values, with p-value <0.05 considered significant.

### Data Availability

Mass spectrometry-derived data have been deposited to the ProteomeXchange Consortium (http://proteomecentral.proteomexchange.org) *via* the PRIDE partner repository (34) with the dataset identifier: PXD015872.

## Results

### PMA-Induced THP-1 Differentiation Causes Changes in Cellular Morphology and Cell-Surface Marker Expression

**Figure 1:**
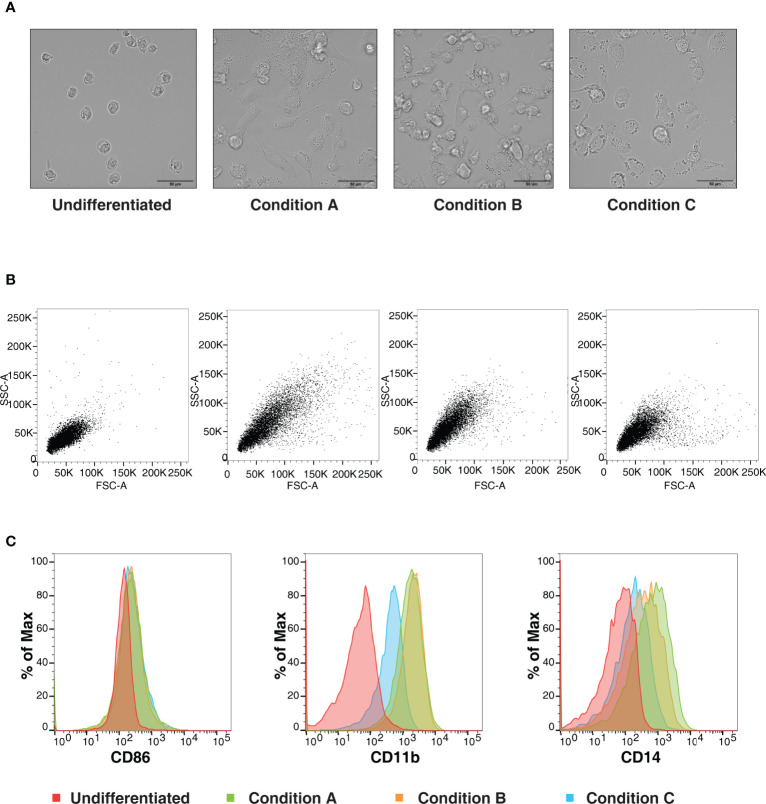
Changes in cell morphology and surface receptor expression are dependent on PMA- mediated differentiation conditions. **(A)** Representative bright-field images (Scale bar:50 μm) **(B)** Representative forward and side light scatter plot **(C)** Representative flow cytometric analysis of THP-1 cells stained using anti- CD86, CD11b or CD14 of THP-1 cells differentiated with varying concentration of PMA (Condition A: 50ng/ml PMA 72h, +48h rest, Condition B: 50ng/ml PMA overnight, + 48h rest and Condition C: 5 ng/ml PMA 48h, +3h rest). Data is representative of at least three independent experiments.

We aimed to investigate the effects of various concentrations and duration of incubation with PMA on the differentiation of THP-1 monocytic cells to macrophage-like cells. We chose three commonly used differentiation protocols for the analysis, as described above. Light microscopy analysis revealed changes in PMA induced morphology, including increased cellular adhesion and spread morphology. In concordance with the results observed by Starr *et al.* (35), the changes in cell morphology were dependent on the concentration and the duration of incubation, with cells treated with PMA and rested for two days showing a significant increase in cytoplasmic volume with increased adherence (**Figure 1A**). The differentiation was more pronounced in Condition A in comparison with the conditions B and C. Additionally; flow cytometry analyses indicated an increase in side scatter (SSC), in condition A with respect to conditions B and C. The cells treated with PMA but not rested (Condition C) closely resembled the undifferentiated THP-1 cells in regard to these properties (**Figure 1B**). Further, the differential expression of cell surface markers CD86, CD11b (ITGAM), and CD14 were monitored. CD86, a cell surface glycoprotein expressed on all antigen-presenting cells, was found to be expressed to a similar extent in all the three protocols tested. In concordance with earlier reports, the expressions of CD11b and CD14 were found to be lower in Condition C in comparison to the other two conditions tested. However, between conditions A and B, the expression was higher in condition A indicating that the duration of incubation and period of rest has an effect on the extent of cell surface expression (**Figure 1C**). Our data, therefore, confirm the previous findings that the degree of differentiation induced by PMA treatment varies depending on the concentration and period of rest post-PMA treatment.

### Quantitative Proteomic Analysis Reveals Diverse Proteome Expression Profiles in Response to Varying Differentiation Protocols

**Figure 2:**
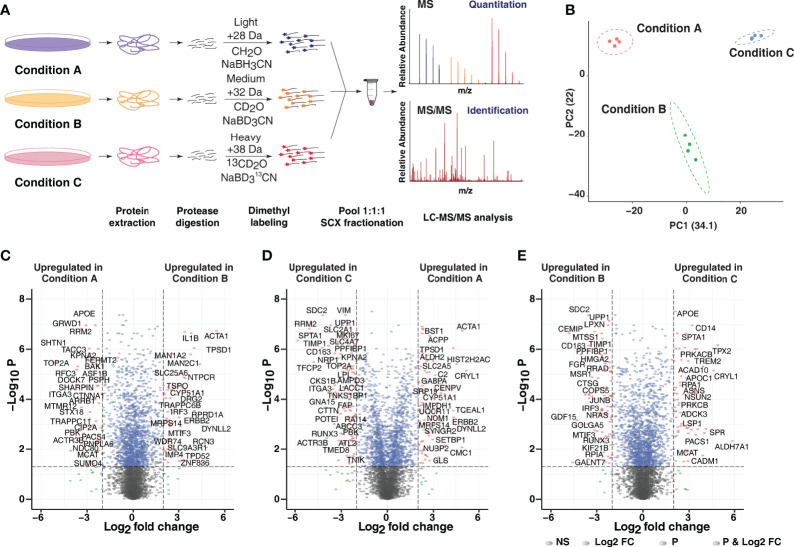
Quantitative proteomic analysis of PMA-induced changes. **(A)** The experimental strategy employed for comparative proteome analysis in response to varying PMA-mediated differentiation protocols. Dimethyl labeling-based quantitative proteomic approach was employed to identify and quantify the proteome changes in Condition A (Light), Condition B (Medium), and Condition C (Heavy). The data were acquired in biological quadruplicates, and only proteins identified and quantified in all 4 replicates were considered for further analysis. **(B)** Principal component analysis (PCA) reveals that the three treatment conditions for monocyte-to macrophage differentiation of THP-1 cells segregate from each other on the basis of concentration and duration of PMA treatment. All replicates of a given condition cluster together, suggesting minimal biological variability. **(C–E)** Volcano plot displaying differential expressed proteins between **(C)** Condition A *vs.* B **(D)** Condition C *vs.* A and **(E)** Condition C *vs.* B. The vertical axis (y-axis) corresponds to the mean expression value of log 10 (p-value), and the horizontal axis (x-axis) displays the log2-fold change value. The red dots represent overexpressed proteins, and the green dots represent proteins with downregulated expression. Positive x-values represent overexpression, and negative x-values represent down-regulation.

To evaluate PMA-mediated proteome-wide expression changes, quantitative proteomic analysis using a stable isotope dimethyl labeling approach was performed (**Figure 2A**). From four independent biological replicates, 5,277 proteins were identified, of which 5,006 proteins were quantified in at least one replicate. A total of 3,623 proteins were identified and quantified in all four replicates providing a global view of changes in protein expression upon PMA treatment (**Supplementary Table 1**). Principal component analysis (PCA) and Spearman correlation matrix revealed distinct clustering of each treatment condition with the biological replicates grouped (**Figure 2B** and **Supplementary Figures 1A, B**). The highest variance was observed in all replicates of condition C, which can likely be explained by the fact that the morphological phenotype observed was much closer to the monocytic cell type rather than the macrophage-like phenotype.

To identify differentially expressed proteins across the three tested protocols, log2 (fold-change) and adjusted p-value cutoff of 2 and < 0.05 was applied. 324, 415 and 413 proteins were found to be overexpressed and 299, 321 and 338 proteins were found to be downregulated in condition B with respect to condition A (B/A), condition C with respect to condition A (C/A) and condition B (C/B), respectively (**Figures 2C–E** and **Supplementary Figure 1A**). The segregation of condition A from condition B and C was driven mainly by differential expression of several proteins involved in vesicle-mediated transport (KIF13B, KIF2C, KIFC1, STX18), cell cycle regulation and mitosis (RRM1 and RRM2), DNA polymerase complex subunits- POLD1 and POLD2, topoisomerase TOP2A, TACC3, and PRKACB. Interestingly, proteins involved in innate immune responses such as TBK1, IRF3, IL1B, scavenger receptor SCARB1, and mitochondrial translation initiation factor MTIF3 were significantly differentially expressed in condition B in comparison to conditions A and C. On the contrary, members of the aldehyde dehydrogenase family (ALDH1L2, ALDH2), members of serine/threonine-protein kinase C (PRKCA, PRKCB, PRKCD), proteins involved in amino acid metabolism-GOT1, PSAT1, ASNS, among others, were found to be significantly upregulated in condition C in comparison to conditions A and B. It has been previously shown that PMA-mediated differentiation increases the expression of PKC isoenzymes, albeit to a varied extent (36). The results from the MS analysis were further confirmed by validating the expression dynamics of select proteins using immunoblot analysis which revealed increased expression in cells differentiated for 3 and 5 days (conditions A and B) (**Supplementary Figure 1C**).

Monocyte-to-macrophage differentiation is reportedly associated with changes in the expression of cell surface proteins, and this phenomenon has been utilized to distinguish macrophage subtypes by their pattern of cell surface receptor expression (7, 37). Our analysis revealed increased expression of known macrophage cell-surface markers such as TFRC (CD71), FCGR1B, scavenger receptors- CD163, MSR1, and SCARB2, mainly in THP-1 cells subjected to conditions A and B. Notably, CD163, a member of the scavenger receptor cysteine-rich (SRCR) superfamily class B, has been previously reported to be highly expressed in macrophages with low expression reported in monocytes, dendritic cells and Langerhans cells (38, 39). On the contrary, the expression of CD68 and CD14, in agreement with previous studies, were downregulated in condition B, suggesting a phenotype closer to macrophages. Interestingly, the expression of CD36 and ITGAM (CD11b) was upregulated in condition A with respect to conditions B and C, whereas that of CD68 was downregulated in both conditions A and B with respect to condition C (**Supplementary Figure 1D** and **Table 1**). Comparison with the results from proteomic analysis of primary monocytes obtained from Rieckmann et al. (40) indicated a moderate degree of similarity/correlation between proteomic profiles of THP-1-macrophages and primary monocytes (**Supplementary Figure 2A**). As reported earlier, the expression of monocyte markers CD14 and CD11b was higher in the primary monocytes compared to the PMA differentiated macrophages (**Supplementary Figure 2B**). On the contrary, classical macrophage markers such as CD71 (TFRC), MSR1, SCARB2, and FCGR1B were found to be selectively upregulated in PMA-differentiated macrophages and not in primary monocytes (**Supplementary Figure 2C**). Given the diversity and heterogenous expression of proteins between primary cells and THP-1 cell lines, expected differences was observed in terms of expression of monocytic/macrophage markers.

**Table 1:** Expression profile of monocyte-macrophage differentiation markers identified in this study.

	Protein description	Gene symbol	Log2 fold change (B/A)	Log2 fold change (C/A)	Log2 fold change (C/B)	P.Value	adj.P.Val
1	Monocyte differentiation antigen CD14	*CD14*	-2.48	0.72	3.20	2.20E-10	1.11E-07
2	Platelet glycoprotein 4	*CD36*	-0.36	-0.06	0.30	0.2006642	0.298214188
3	Macrosialin	*CD68*	-0.48	0.75	1.22	0.0130009	0.03372904
4	Transferrin receptor protein 1	*TFRC* (CD71)	-0.78	-1.82	-1.04	3.92E-08	2.59E-06
5	Scavenger receptor cysteine-rich type 1 protein M130	*CD163*	-0.30	-3.83	-3.53	5.45E-09	7.21E-07
6	High affinity immunoglobulin gamma Fc receptor IB	*FCGR1B*	-0.03	-0.51	-0.48	0.4016242	0.50809358
7	Macrophage scavenger receptor types I and II	*MSR1*	-0.41	-2.52	-2.11	1.93E-07	7.16E-06
8	Scavenger receptor class B member 1	*SCARB1*	3.24	0.64	-2.59	1.63E-06	3.38E-05
9	Lysosome membrane protein 2	*SCARB2*	-0.74	-1.15	-0.41	2.50E-07	8.23E-06
19	Integrin alpha-L	*ITGAL* (CD11a)	0.29	1.33	1.04	1.83E-08	1.56E-06
11	Integrin alpha-M	*ITGAM* (CD11b)	-0.92	-0.95	-0.03	1.95E-07	7.16E-06
12	Integrin alpha-X	*ITGAX* (CD11c)	-0.50	-0.42	0.08	0.0082739	0.023734127

### Cluster Analysis Reveals Differentiation Protocol-Specific Regulation of Cellular Processes and Signaling Pathways

**Figure 3:**
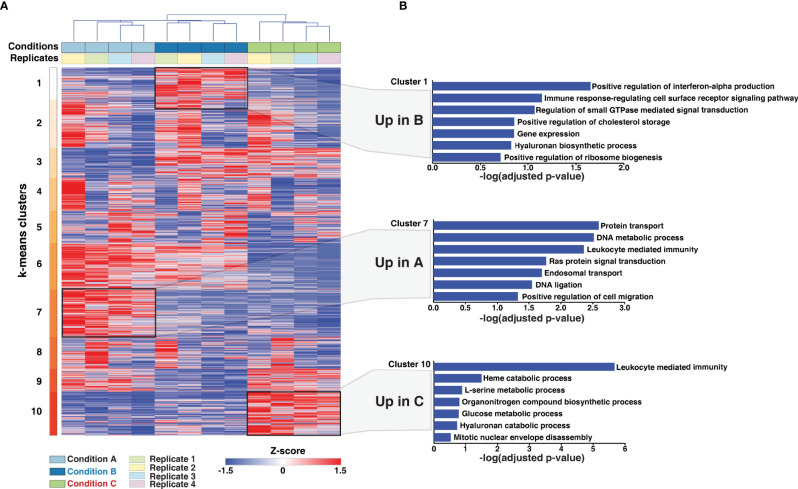
Protein expression dynamics analysis upon PMA induced monocyte to macrophage differentiation. **(A)** Protein expression patterns in response to differentiation protocols A-C were analyzed. Log2 transformed, z-score normalized and scaled expression of proteins identified and quantified in all replicates were plotted, and k-means clustering was carried out. Clusters 1, 7, and 10 represent overexpressed proteins exclusive to conditions A-C, indicating induction of differentiation-protocol-specific proteome signatures. **(B)** The proteins exclusive to each condition were subjected to Gene Ontology (GO) analysis using Enrichr to understand their function. Selected significantly enriched GO terms (biological processes) (p-value<0.005) have been highlighted. Notably, proteins involved in several metabolic processes were largely enriched in Condition C compared to the other two conditions tested. Overall, the primary cellular process of macrophages, i.e., mediating immune response is enriched in all three conditions tested.

Using Euclidean average and k-means clustering, the differentially expressed genes were segregated into 10 major clusters (**Figure 3A** and **Supplementary Table 2**). A high correlation between biological replicates was observed across the quantified proteins. Cluster 1, 7, and 10 included proteins upregulated in conditions B, A, and C, respectively. 290 proteins (Cluster 3) were expressed to a similar extent in conditions B and C but downregulated in condition A. On the contrary; 458 proteins (Cluster 6) were expressed to a similar extent in conditions A and B but downregulated in condition C. Cluster 9 comprised of proteins overexpressed in conditions A and C in comparison to condition B, but with an overall increased expression observed in condition C. Clusters 2, 4 and 5 showed similar expression of proteins across all three protocols tested with varying expression across replicates. Altogether, our analysis demonstrates the existence of universal and differentiation-protocol-specific proteome signatures.

Gene ontology analysis of the differentiation protocol-specific clusters (clusters 1, 7, and 10) revealed significant enrichment of several biological processes (adjusted p-value < 0.05) (**Figure 3B**, **Supplementary Tables 3**–**5**) including metabolic processes and processes indicative of differentiation, such as cell migration and mitotic nuclear envelope assembly. While immune response-regulating cell surface receptor signaling pathway was observed to be primarily enriched in condition B, the process of leukocyte mediated immunity was found to be enriched in conditions A and C (**Supplementary Figure 3**). Regulation of translation and gene expression were among the significantly enriched processes in Cluster 3 and 6. In contrast, carbohydrate and fatty acid metabolic processes were significantly enriched in Cluster 9, which comprises of proteins expressed to a similar extent in conditions A and C but downregulated in condition B (**Supplementary Figure 3**). Overall, the findings are consistent with the morphological changes observed with processes indicative of differentiation, such as cell migration, gene expression, ribosome biogenesis as well as immune cell function, primarily enriched in conditions A and B, respectively.

### Pathway Enrichment and Network Analysis Reveals Kinases as Key Regulatory Hubs of the PMA Mediated Differentiation Processes

**Figure 4:**
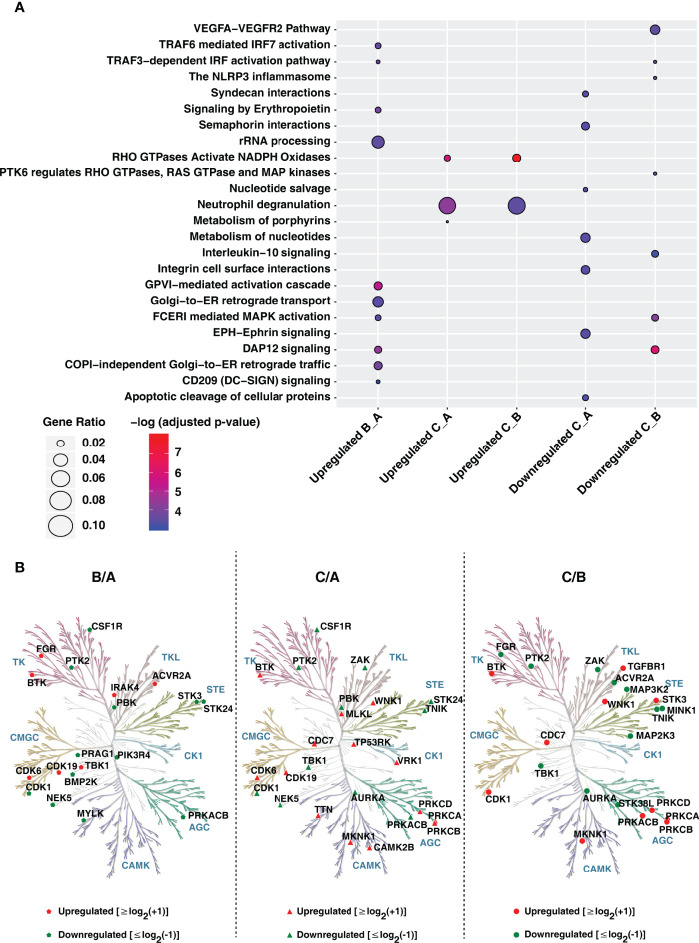
Gene ontology analysis of proteins upon PMA induced monocyte to macrophage differentiation. **(A)** Gene Ontology analysis of upregulated and downregulated proteins in conditions A-C using ReactomePA. The X-axis represents proteins changing in Condition B with respect to A (B_A), Condition C with respect to A (C_A) and Condition C with respect to B (C_B). **(B)** Kinome trees showing differential regulation of protein kinases in response to differentiation conditions A-C. The phylogenetic kinase relationship (47) were generated using KinMap. Protein kinases identified and quantified in our study are indicated as red (upregulated) and green (downregulated) symbols (circles and triangles), respectively.

We next aimed to delineate the signaling pathways affected during monocyte-to-macrophage differentiation. Pathway enrichment analysis using the Reactome database revealed differentiation protocols specific enrichment of signaling pathways such as the VEGF signaling pathway (enriched in conditions A and B), metabolism of nucleotides and porphyrins (enriched in condition A), Fc epsilon R1 signaling (enriched in condition B), activation of NADPH oxidases by RhoGTPases, and glycosphingolipid metabolism (enriched specifically in condition C) (**Figure 4A** and **Supplementary Table 6**). Interestingly, cell surface signaling mediated by ephrins, integrin, semaphorin, and syndecan interactions were significantly enriched in condition A with respect to condition C with no apparent differences observed with respect to condition B. On the contrary, signaling pathways involved in clearing infections and immune responses were significantly enriched in condition B. Apoptotic pathways were found to be downregulated. In contrast, transamination and amino acid synthesis were upregulated in condition C.

Network analyses of k-means clusters upregulated in each condition (**Supplementary Figures 4**–**6** and **Supplementary Table 7**) enabled the identification of distinct hub proteins. Several kinases involved in cellular processes, namely cell proliferation, differentiation, and regulation of microtubule dynamics such as MAPK1, CDK1, and PRKACB, were found to have a high degree of betweenness centrality and exist as key regulatory hubs in the network for condition A. Other vital regulatory hubs included DUT, FN1 a glycoprotein involved in cell adhesion and migration processes, and SEC13, a core component of the COPII-coated vesicles and nuclear core complex. While proteins involved in innate immune response such as ISG15, IL1B, LYN, COPS5, TBK1, MRPL3, and KRAS were found to be critical regulatory hubs in condition B, enzymes involved in amino acid metabolism such as GOT1, PSAT1, AARS, CBS, IMPDH1; subunit of RNA polymerase II (POLR21), plasma membrane-associated Rho GTPase RAC1 and PRKCD were found to critical regulatory proteins in condition C. Interestingly, a previous study exploring the role of kinases in monocyte-macrophage differentiation observed a pronounced decrease in the expression of regulatory kinases such as CDK1 involved in cell cycle entry and checkpoint in PMA-differentiated THP-1 macrophage-like cells (22). It is well known that PMA activates protein kinase C (PKC) and promotes leukocyte adhesion and migration, and therefore identifying PRKCD as one of the regulatory hubs is indicative of the monocyte-to-macrophage differentiation process.

Further investigation on the effect of PMA differentiation protocols on the extent of expression of other protein kinases as well as phosphatases revealed increased expression of several kinases belonging to diverse classes (**Figure 4B**). Notably, protein tyrosine kinases CSF1R and PTK2 were overexpressed in condition A, FGR, a member of the c-Src family tyrosine kinases known to be induced by PMA (41–43) in condition B, and BTK2 in condition C, respectively. Interestingly, increased expression of several kinases was observed in condition C, including members of the protein kinase C family-PRKCB, PRKCD which are known markers of immune cell differentiation and inflammation, MAP kinase-interacting serine/threonine-protein kinase 1 (MKNK1), MAPK14 and WNK1, a known regulator of ion transport proteins involved in the differentiation and migration of endometrial stromal cells (44) and glioma cells (45). Among the protein phosphatases, 80 were identified and quantified in our dataset, with a vast majority expressed to a similar extent in all conditions tested. Of note, phosphatases belonging to the HP2 family namely: prostatic acid phosphatase ACPP and acid phosphatase 2 (ACP2), were significantly dysregulated in expression with ACPP over 20-fold overexpressed in condition C with a similar level of expression observed in conditions A and B. On the contrary, ACP2, a lysosomal acid phosphatase was overexpressed 2-fold higher in condition A compared to both condition B and C. Among the dual-specificity protein phosphatases (DUSPs), SSH3, a member of the slingshot family was upregulated in condition C. SSH3 is known to specifically dephosphorylate and activate Cofilin, one of the key regulators of actin filament dynamics and remodeling (46). Of the 16 protein tyrosine phosphatases (PTPs) identified, the expression of PTPN7, PTPRC were high in condition C whereas PTPN12, PTPN23, PTPN2, PTPRA, PTPRK, and PTPRU were overexpressed in condition A. Interestingly, PTPRE and to a smaller extent, PTPN6 demonstrated increased expression in condition B. Collectively, the protein kinases and phosphatases identified in this study indicate their differential capacity in the regulation of microtubule stability, actin cytoskeleton reorganization and autophagy in macrophages which in turn are required for their functional responses including phagocytosis, antigen presentation, DAMP and PAMP-mediated immune signaling.

### PMA-Induced Monocyte-to Macrophage Differentiation Modulates the Expression Dynamics Proteins Involved in Innate Immune Signaling

**Figure 5:**
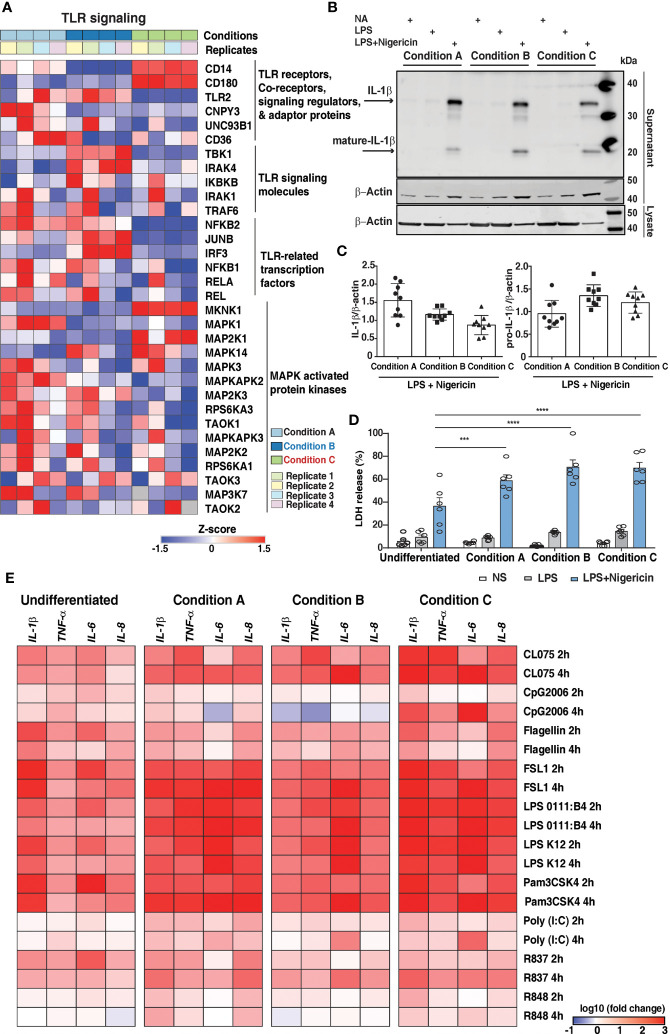
Expression dynamics of genes involved in innate immune signaling in response to PMA. **(A)** Heatmap demonstrates the proteome fold change of a subset of genes involved in innate immune signaling. The scale indicates the level of expression (Log2-expression values, z-transformed, scaled). **(B)** Representative image of Immunoblot analysis and **(C)** quantification by densitometry of pro- and mature IL-1β protein in supernatants of PMA differentiated THP1 cells treated with LPS (2h) followed by NLRP3 agonist; nigericin (2h). Blots are representative of 3 independent experiments analyzed in technical triplicates. Data are shown as mean ± SEM (two-tailed Student’s t-test) **(D)** LDH release assay of PMA differentiated THP1 cells (n=6) treated with LPS (2h) followed by NLRP3 agonist; nigericin (2h). (measured as % LDH release of total lysis control). Data are shown as mean ± SEM. ***P 0.0003, ****P<0.0001 by one-way ANOVA with Tukey’s multiple-comparisons test **(E)** Dynamic expression pattern to assess the expression levels of pro-inflammatory cytokine mRNA induced by TLR agonists for 2h and 4h: CL075-TLR7/8, CpG2006-TLR9, Flagellin-TLR5, FSL1-TLR2/6, LPS 0111:B4, and LPS K12 – TLR4, Pam3CSK4-TLR2/1, Poly I:C-TLR3, R837-TLR7, and R848-TLR7/8. Heatmap depicts the Log10 fold change of normalized gene expression for pairwise comparisons of mRNA levels.

We next aimed to assess the impact of PMA-mediated differentiation protocols on the expression of proteins involved in innate immune signaling as THP-1 cells are widely used as *in vitro* model to study immune modulatory effect. Analysis of the expression profile of proteins involved in Toll-like receptor (TLR) signaling pathways revealed differences in the expression of known downstream effector proteins (**Figure 5A** and **Supplementary Table 8**). A majority were expressed at similar levels in all three conditions, suggesting that the effect of following secondary inflammatory stimuli would be mostly independent of PMA stimulation. Among the TLR receptors, we identified only TLR2 with over 2-fold expression in conditions A and B. TLR4 co-receptors, namely CD14 and CD180, were found to be expressed relatively lower extent in conditions A and B as expected. The other signaling regulators and adaptor proteins such as CNPY3, UN93B1, and CD36 were in general upregulated in condition A in comparison to conditions B and C, respectively.

Signaling mediated by TLRs requires the assembly of the Myddosome complex. Although we identified Myd88 (48), quantitation was obtained in only conditions A and B. The other components including IRAK1, IRAK4 and TRAF6, were overexpressed in condition B and condition A compared to condition C. Of the transcription factors essential for mediating TLR activity, increased expression were observed in mostly conditions A and B with JUNB, highly expressed in condition B. RelB, a member of NF-κB family, was identified in only conditions A and B (49, 50). The expression of Interferon regulatory transcription factors also varied across the conditions tested with over 4-fold expression IRF3 expression in condition B in comparison to conditions A and C, whereas IRF5, a key regulator of the antiviral immune response, was 2-fold overexpressed in condition C with respect to conditions A and B. This indicates that using condition C to study TLR signaling and/or non-canonical NF-κB signaling pathways may likely alter the outcome of the experiment. The subsequent phenotype will mostly be dependent on the PMA stimulation. The expression of kinases belonging to the MAPK family, as described in the earlier section, was generally higher in conditions A and B with the exception of MKNK1, MAP2K1, and MAPK14, which were observed to be upregulated in condition C (**Figure 5A**). Our results are in concordance with a previous report suggesting rewiring of MAPK signaling cascade upon THP-1 differentiation (22). We also observed differential expression of proteins known to be involved in the process of phagocytosis and oxidative stress such as FGR, LYN, and pro-inflammatory cytokine IL1B, among others, which were selectively upregulated in condition B. However, neutrophil cytosolic factors NCF2 and NCF4-regulatory components of the superoxide-producing phagocyte NADPH-oxidase, PYCARD, ITGAL, protein kinase C delta (PRKCD), PTPRC, were found to be upregulated in condition C (**Supplementary Figure 7**).

Five proteins known to be a part of the inflammasome complex were identified in this study with similar levels of expression except for NLRP3 that was identified only in condition B. We also observed differences in other proteins related to inflammasome functions such as PYCARD (ASC) and Caspase-1 that are predominantly expressed in condition C and Gasdermin-D in condition A (**Supplementary Figure 7**). Based on our findings, we sought to investigate the effects of PMA differentiation on inflammasome activation in THP-1 macrophages. PMA-differentiated THP-1 cells primed with LPS for 2 hours, followed by treatment with NLRP3 agonist nigericin, resulted in differentiation-dependent variations in IL1β and LDH release, used as a measure of pyroptosis (**Figures 5B–D**). Our results from three independent experiments indicate that both full-length and cleaved IL1β are released upon treatment with LPS and nigericin in all three conditions. Notably, nigericin-induced secretion of full-length IL1β was highest in condition A compared to conditions B and C. On the other hand, the release of mature IL1β in the supernatants was increased in condition B than in conditions A and C, respectively (**Figures 5B, C**). Additionally, the extent of LDH release as expected was the highest upon treatment with LPS and nigericin in all the three conditions tested (**Figure 5D**). Interestingly the LDH release was higher in condition B compared to the other two conditions tested, correlating with an increase in mature IL1β release. Overall, our results suggest that inflammasome activation appears to be pronounced in Condition B compared to the other two tested conditions.

### PMA-Induced Monocyte-to Macrophage Differentiation Modulates the Pro-Inflammatory Response

The response of activated macrophages by various stimuli involves the secretion of cytokines such as IL1β, IL6, IL8, and TNFα as a significant component of the innate immune response (51). We, therefore, studied the functional properties, cytokine gene expression in undifferentiated (monocytic), and differentially differentiated THP-1 cells (macrophage-like) and compared the dissimilarity and similarity of expression trends among conditions. To assess the changes in the cytokine mRNA expression levels, cells were treated with various TLR ligands: CL075-TLR7/8, CpG2006-TLR9, Flagellin-TLR5, FSL1-TLR2/6, LPS 0111:B4, and LPS K12 – TLR4, Pam3CSK4-TLR2/1, Poly I: C-TLR3, R837-TLR7, and R848-TLR7/8 (**Figure 5E**) for 2 hours and 4 hours, respectively. Monocytes are the first cells that encounter pathogens and promptly adapt to the environmental situations by regulating the expression of genes activated by the inflammasome, such as IL1β (52). As anticipated, the TLR2 ligands Pam3CSK4 and FSL-1-stimulated undifferentiated cells showed significant induction of *IL1β* mRNA at both timepoints. Interestingly, they also induced a high level of *IL6* and, to a considerable extent, *IL8* mRNA at an earlier time point (2 hours). On the other hand, the average expression of the cytokines appeared to increase in PMA-differentiated cells with respect to the undifferentiated cells, except when it was simulated with CpG2006 and R837 at an earlier time point (2 hours). Expression of cytokines upon stimulation with FSL1, LPS 0111: B4, LPS K12, and Pam3CSK4 did not show any significant variation among the differentiation conditions except for *IL6* mRNA which was downregulated upon sustained stimulation with CpG2006, Flagellin and R848 stimulation in condition A. We also observed marked downregulation of *IL1β* and *TNFα* mRNA upon 4 hours of CpG2006 stimulation in Condition B. On the contrary, the expression of both cytokines were significantly higher in Condition C. Overall, the most robust upregulation, especially with respect to *IL1β*, *IL6* and *TNFα* expression levels were observed in condition C. Our results are in concordance with previous report (12) indicating that the PMA differentiation protocols modulate the response to secondary stimuli and the extent of induction of pro-inflammatory cytokines by various TLR agonists vary across the three different protocols tested (**Figure 5E**).

Multiplexed cytokine profiling of the supernatants after 8 hours in response to TLR activation strongly indicated that the differences in the gene expression profiles are also reflected in the cytokine production and release. Overall, the extent of cytokine release was more remarkable in PMA differentiated cells than undifferentiated cells. Furthermore, the extent of release was primarily pronounced in conditions B and C except for IL6 and TNF in condition A upon TLR2 activation. Contrary to the IL1β expression induced by TLR agonists in condition B, we observed a significant increase in the release of IL1*β* in response to TLR7/8 (CL075), TLR7 (R837), and TLR4 activation (**Supplementary Figure 8** and **Supplementary Table 9**). While TLR2 ligands were more potent in driving *IL1β*, *IL6*, and *IL8* gene expression in undifferentiated cells, the extent of cytokine release was minimal. Similarly, most TLR agonists induced robust *IL6* gene expression in Condition C; however, the extent of its release was minimal except for TLR4 activated cells. On the contrary, the extent of *IL8* expression and release were almost similar in conditions B and C in comparison to condition A. Altogether, our analyses indicate that depending on the context of the experimental question, careful consideration of the differentiation protocols selection must be made to avoid undesired outcomes.

## Discussion

Human monocytic THP-1 cells are extensively used as a model system to study monocyte/macrophage functions. Nevertheless, to be used as an *in vitro* model mimicking human macrophages, THP-1 cells have to be differentiated, and several protocols have been tested (7, 9, 36, 53). Among these, PMA is most often employed to induce differentiation, with almost similar phenotypes to primary MDMs reported in terms of cell morphology, expression of macrophage surface markers, and cytokine production (35, 54). However, careful consideration and optimization of the timing and dosage of PMA are required as they can influence the differentiation process, and in turn, the phenotypic processes tested. Although studies have described differentiation protocol-mediated changes in the transcriptome profile or surface receptor expression (11, 37), a systematic comparison at the global proteome level has not been reported to date. Towards this end, we aimed to delineate the changes mediated by varying doses and duration of PMA treatment to induce differentiation and its effect on secondary stimuli’ response. Three established and widely employed PMA-mediated differentiation protocols, 5ng/ml and 50 ng/ml PMA treated for 16-72 hours, followed by a resting period ranging from 3 hours- 2 days, were considered. Using an unbiased high throughput quantitative proteomic approach, our results highlight the proteome difference of the differentiated cells, and the effect of inflammatory stimuli further validates that the differentiation process affects the phenotype under investigation.

THP-1 cells treated with 50ng/ml PMA and rested for two days (Conditions A and B) appeared to correlate well with macrophage-like phenotype both in terms of morphological characteristics, cell surface expression of markers, and proteome profiles, unlike what was suggested previously (12). Importantly, cells treated with 50ng/ml PMA overnight and rested for 2 days (Condition B) was sufficient to induce differentiation as determined by reduced expression of monocyte markers CD14 and CD11b. Furthermore, the expression of classical macrophage markers such as CD71 (TFRC), MSR1, FCGR1B and CSF1R was upregulated without overtly increased expression as observed in cells treated with the same dose of PMA but for 72h and rested for 2 days (Condition A). A moderate degree of similarity/correlation between proteomic profiles of THP-1-macrophages and primary monocytes was also observed upon the comparison of our results with proteomic data on primary monocytes (Pearson correlation coefficient~0.5) (40). Given the diversity and heterogeneous expression of proteins between primary monocytes and THP-1 cell lines, expected differences were observed in terms of expression of monocytic/macrophage markers.

Furthermore, we delineated the biological and cellular processes that are impacted to a considerable extent depending on the type of treatment conditions. Notably, regulation of immune signaling response, protein transport, and regulation of cell migration, all indicative of macrophage function, were significantly enriched in conditions A and B. Our findings are in concordance with previous study on global transcriptome analysis of GM-CSF-induced macrophages (55). The increased expression of *IL1β* upon PMA differentiation has been reported earlier at the transcriptome level and we observed distinct upregulation in condition B in comparison with other conditions further validating our results (11). Although we did not examine the phagocytic activity of differentiated macrophages in this study, we provide evidence of differential expression of proteins involved in the process of phagocytosis across the three tested protocols. Our analysis further highlights differentiation protocol-specific subsets with several protein kinases serving as central hubs in mediating the differentiation process. This is of vital importance as cellular signaling is primarily governed by the regulation of expression of kinases and phosphatases in a cell type-specific manner or upon cell activation (32, 40, 56). Key regulatory kinases implicated in cell cycle regulation including cyclin-dependent kinases and NEK family of serine-threonine kinases were found to be dysregulated and is in concordance with previous study exploring the role of kinases in monocyte-macrophage differentiation (22). Similarly, a moderate increase in the expression of MAPKs in conditions A and B was observed, likely indicating rewiring of the MAPK-signaling cascades upon monocyte to macrophage differentiation as described previously (22).

Studies have shown that altered sensitivity and undesirable gene regulation in PMA-differentiated macrophages may contribute to their differential response to secondary stimuli (12, 35). Although we observed differential expression of proteins involved in innate immune signaling across the three protocols, a majority demonstrated a mixed expression pattern. To determine whether the protein expression dynamics influenced the response to secondary stimuli, we assessed two aspects of innate immune signaling, namely inflammasome activation and response to TLR agonists. Inflammasome activation is a vital process required for the initiation of an innate immune response upon exposure pathogen-associated molecular patterns (PAMPs) and danger-associated molecular patterns (DAMPs) (57–59) resulting in the release of pro-inflammatory cytokines such as IL1β and IL18, ultimately leading to pyroptosis (60). Examining the effects of primary and secondary innate immune stimuli (LPS and LPS plus nigericin) on the three PMA differentiation protocols interestingly revealed an increased release of LDH and the mature form of IL1β in condition B. The relevance and specific mechanisms behind this phenomenon, however, remains to be determined. Concerning cytokine responses induced in monocytes and macrophages, the extent of production and release vary depending on the stimulus and the type of differentiated macrophages. Our findings encompassing gene expression analysis and cytokine profiling indicate a time-dependent increase in the transcriptional response of pro-inflammatory cytokines upon stimulation with TLR ligands except for R848- a TLR8 agonist and CpG2006, a TLR9 agonist. Furthermore, the release of pro-inflammatory cytokines in response to TLR activation strongly indicated that the differences in the gene expression profiles are also reflected in the cytokine production and release. Interestingly, we note that the release of IL1β was observed to be higher in condition B in response to TLR4 and TLR7 activation. Taken together, though the three differentiation conditions show similarities in innate immune and inflammatory responses, the extent of these responses across the three conditions vary to a considerable extent, with condition B showing optimal responses across the characteristics tested. Therefore, the use of condition B for THP-1 differentiation could provide optimum conditions to study inflammasome activation as well as the innate immune response.

## Conclusions

Minor deviations in differentiation protocols can have unintended effects on the overall experimental setup and the results. Therefore, validation of the model solely through morphological or cell surface expression of select markers may not suffice and should be supplemented with orthogonal experiments that provide a more significant overview of the cellular state. Using an integrated approach, we demonstrate the impact of differentiation protocols on the cellular proteome, biological processes and its potential to alter immunological responses in PMA differentiated macrophage like cells as reference cell model. Employing the most relevant biological state will thus be fundamental to generate meaningful and interpretable results.

## Data Availability Statement

The datasets presented in this study can be found in online repositories. Mass spectrometry-derived data have been deposited to the ProteomeXchange Consortium (http://proteomecentral.proteomexchange.org) *via* the PRIDE partner repository (34) with the dataset identifier: PXD015872.

## Author Contributions

SP, HK, MG, KB, and LR performed experiments. RK conceived the study. SP, HK, YS, MG, KB, and AS analyzed data. SP, HK, KB, and RK designed the experiments. RK supervised the bioinformatics analysis and follow-up validations. RK, SP, HK, and YS wrote the manuscript. All authors contributed to the article and approved the submitted version.

## Funding

This research was funded by the Research Council of Norway (FRIMEDBIO “Young Research Talent” Grant 263168 to RK; and Centres of Excellence Funding Scheme Project 223255/F50 to CEMIR), Onsager fellowship from NTNU (to RK), and Regional Health Authority of Central Norway (90414000 to KB).

## Conflict of Interest

The authors declare that the research was conducted in the absence of any commercial or financial relationships that could be construed as a potential conflict of interest.

## Abbreviations

BCA, Bicinchoninic Acid; DTT, Dithiothreitol; TEABC, Triethyl ammonium bicarbonate; FACS, Fluorescence-Activated Cell Sorting; GO, Gene Ontology; HCD, Higher-energy Collisional Dissociation; IAA, Iodoacetamide; MS/MS, Tandem mass spectrometry; PCA, Principal Component Analysis; PMA, Phorbol-12-myristate-13- acetate; SCX, Strong Cation Exchange; TLR, Toll-Like Receptor; UHPLC, Ultra-High-Performance Liquid Chromatography.
