# Supplementary material for: Comparative Proteomic Analysis Reveals Varying Impact on Immune Responses in Phorbol 12-Myristate-13-Acetate-Mediated THP-1 Monocyte-to-Macrophage Differentiation
Source: Front Immunol. 2021 Jun 21;12:679458. doi: 10.3389/fimmu.2021.679458 (PMC8255674; doi:10.3389/fimmu.2021.679458)
Supplement: Supplementary file 10 [file Image_1.pdf]

Comparative proteomic analysis reveals varying impact on immune responses in phorbol 12-myristate-13-acetate-mediated THP-1 monocyte-to-macrophage differentiation

Sneha M. Pinto[1,2*], Hera Kim[1*], Yashwanth Subbannayya[1], Miriam Giambelluca[1], Korbinian Bösl[1,3] Liv Ryan[1], Animesh Sharma[4], and Richard K. Kandasamy[1, #]

[1]Centre of Molecular Inflammation Research (CEMIR), and Department of Clinical and Molecular Medicine (IKOM), Norwegian University of Science and Technology, 7491 Trondheim, Norway

[2]Center for Systems Biology and Molecular Medicine, Yenepoya (Deemed to be University), Mangalore, India

[3]Department of Infectious Diseases, Medical Clinic, St. Olavs Hospital, 7491 Trondheim, Norway

[4]Proteomics and Modomics Experimental Core, PROMEC, at NTNU and the Central Norway Regional Health Authority, Stjørdal, Norway

* These authors contributed equally

# Correspondence:
Richard K. Kandasamy
richard.k.kandasamy@ntnu.no

Supporting Information
Figures S1-S8
Tables S1-S9

Supplementary Figure legends

Supplementary Figure 1

(A) Principal component analysis (PCA) based on the proteins driving the segregation between the monocyte-to-macrophage differentiation protocols. Proteins highlighted in blue, purple, and red segregate conditions B, A, and C, respectively.

(B) Similarity matrix of protein expression in the three tested differentiation protocols. The correlation was carried out using Spearman's rank correlation, which good correlation among the replicates.

(C) Western blot analysis of THP-1 cells differentiated with various concentrations of PMA in comparison with undifferentiated THP-1 monocytes using antibodies against IRF3, TBK1, IL1B, and SQSTM1. Immunoblot analysis was performed in triplicates. GAPDH was used as a loading control.

(D) Heatmap depicting cell surface receptor expression across the three differentiation protocols. The scale indicates the level of expression (Log2-expression values, z-transformed, scaled

Supplementary Figure 2

(A) Correlation matrix between proteomic profiles of THP-1 cells differentiated using three different conditions- A, B, C, and primary monocyte data obtained from Rieckmann *et al.*

(B, C) Histograms showing expression of canonical monocyte and macrophage markers in THP-1 cells differentiated using three different conditions- A, B, C, and primary monocyte data obtained from Rieckmann *et al.*

Supplementary Figure 3

Significantly enriched biological processes (p-value$\leqslant$0.005) for K-means clusters 3, 6, and 9. Heatmap depicts the expression changes of selected genes associated with the respective biological process.

Supplementary Figure 4

Network analysis of proteins upregulated in condition A. The network properties were calculated, and the betweenness centrality and degree measures have been indicated using node size and color, respectively. Sub clustering of network yielded functional clusters and have been depicted.

Supplementary Figure 5

Network analysis of proteins upregulated in condition B. The network properties were calculated, and the betweenness centrality and degree measures have been indicated using node size and color, respectively. Sub clustering of network yielded functional clusters and have been depicted.

Supplementary Figure 6

Network analysis of proteins upregulated in condition C. The network properties were calculated, and the betweenness centrality and degree measures have been indicated using node

size and color, respectively. Sub clustering of network yielded functional clusters and have been depicted.

Supplementary Figure 7

Dynamic expression pattern to assess the expression levels of proteins components of the inflammasome complex, ROS markers, and phagocytosis. The scale indicates the level of expression (Log2-expression values, z-transformed, scaled).

Supplementary Figure 8

Differential cytokine secretion after stimulation with TLR agonists. Undifferentiated THP-1 cells (U) and THP-1 cells differentiated with the various PMA concentrations (A, B, C) were stimulated as described in Figure 5E, and the supernatant was collected after 8 h and analyzed by multiplex bioassay (n= 3). As observed in Figure 5E, the differences in gene expression are also evident in cytokine production and release.

Supplementary Figure 1

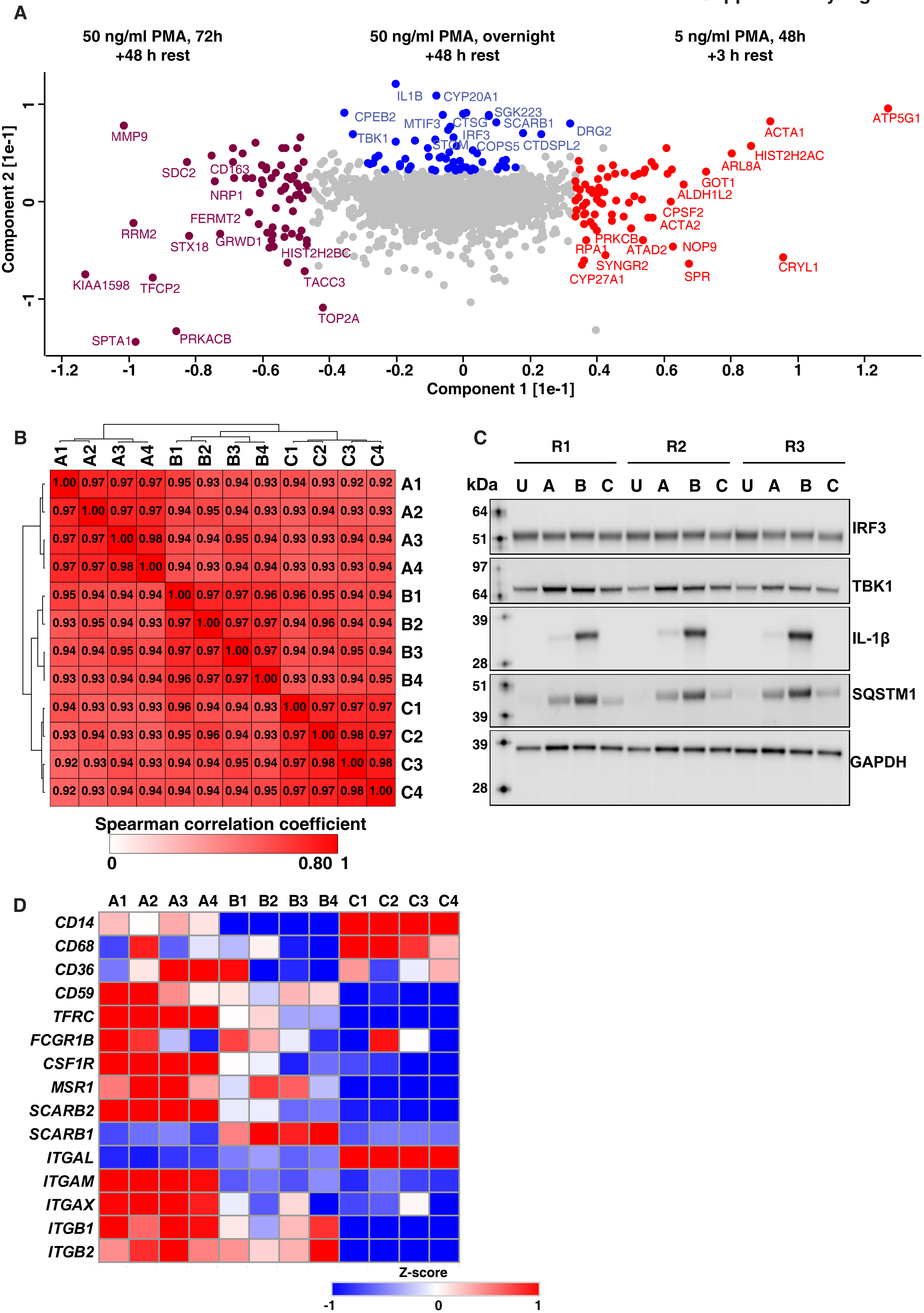

Supplementary Figure 2

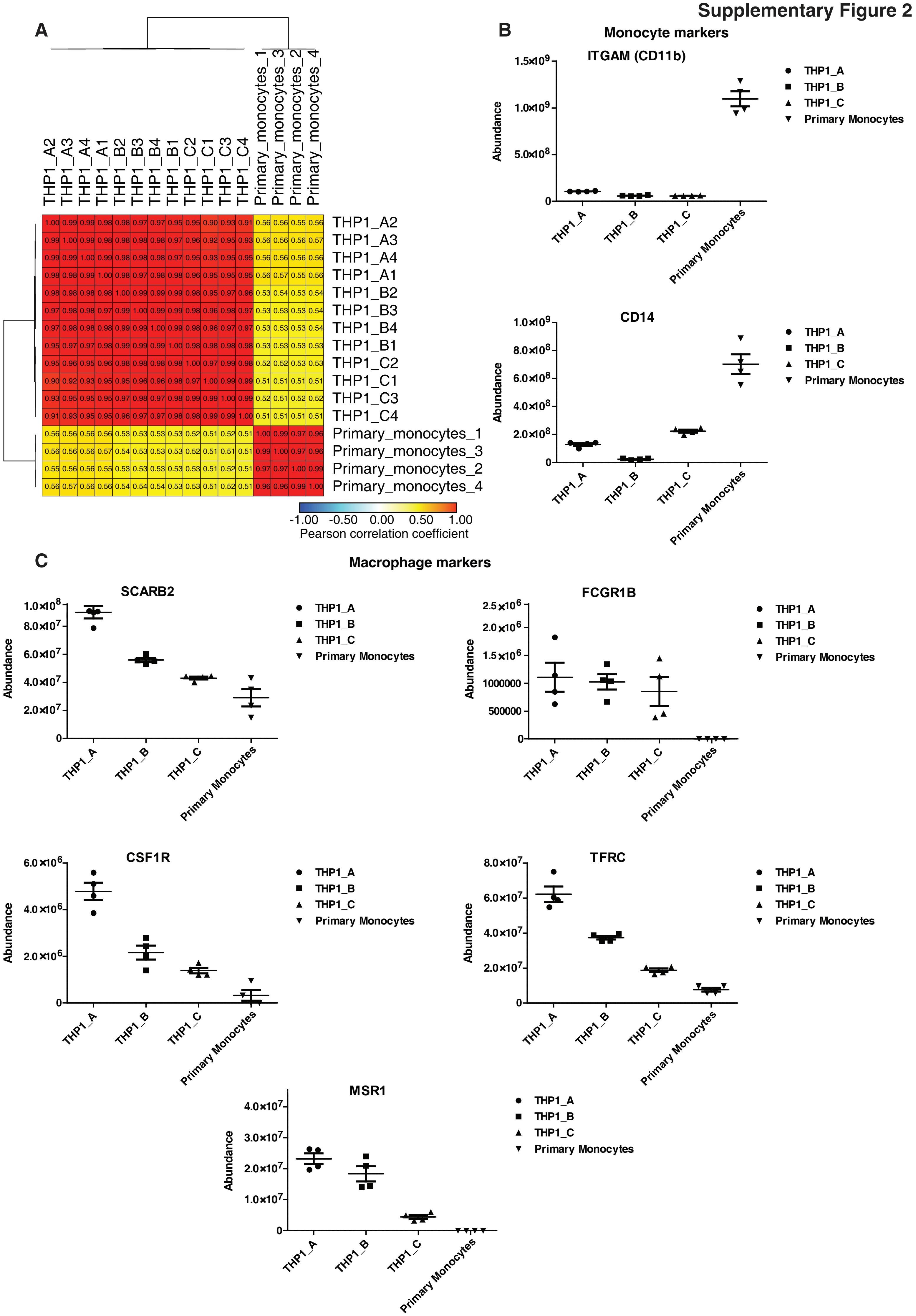

Supplementary Figure 3

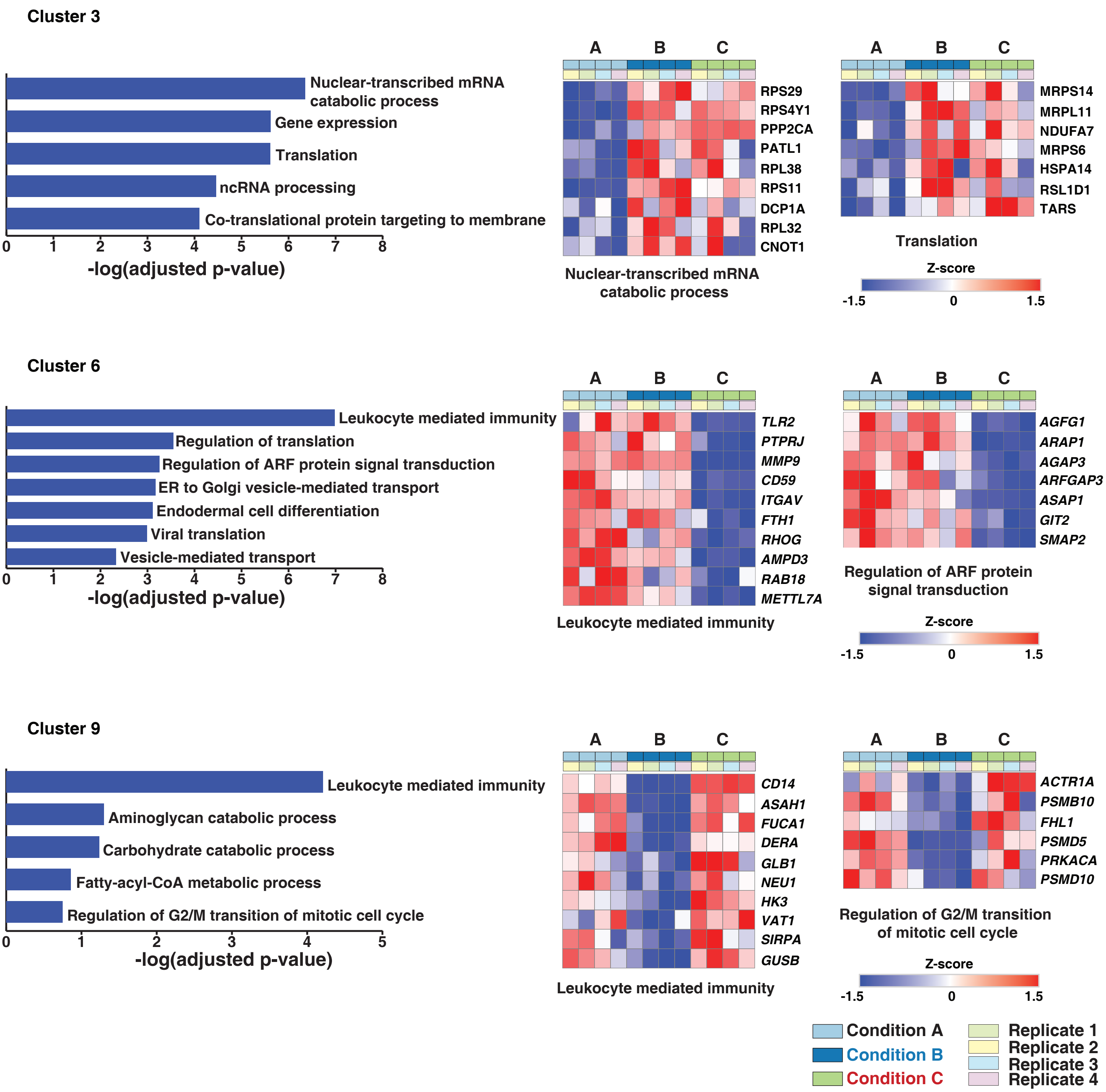

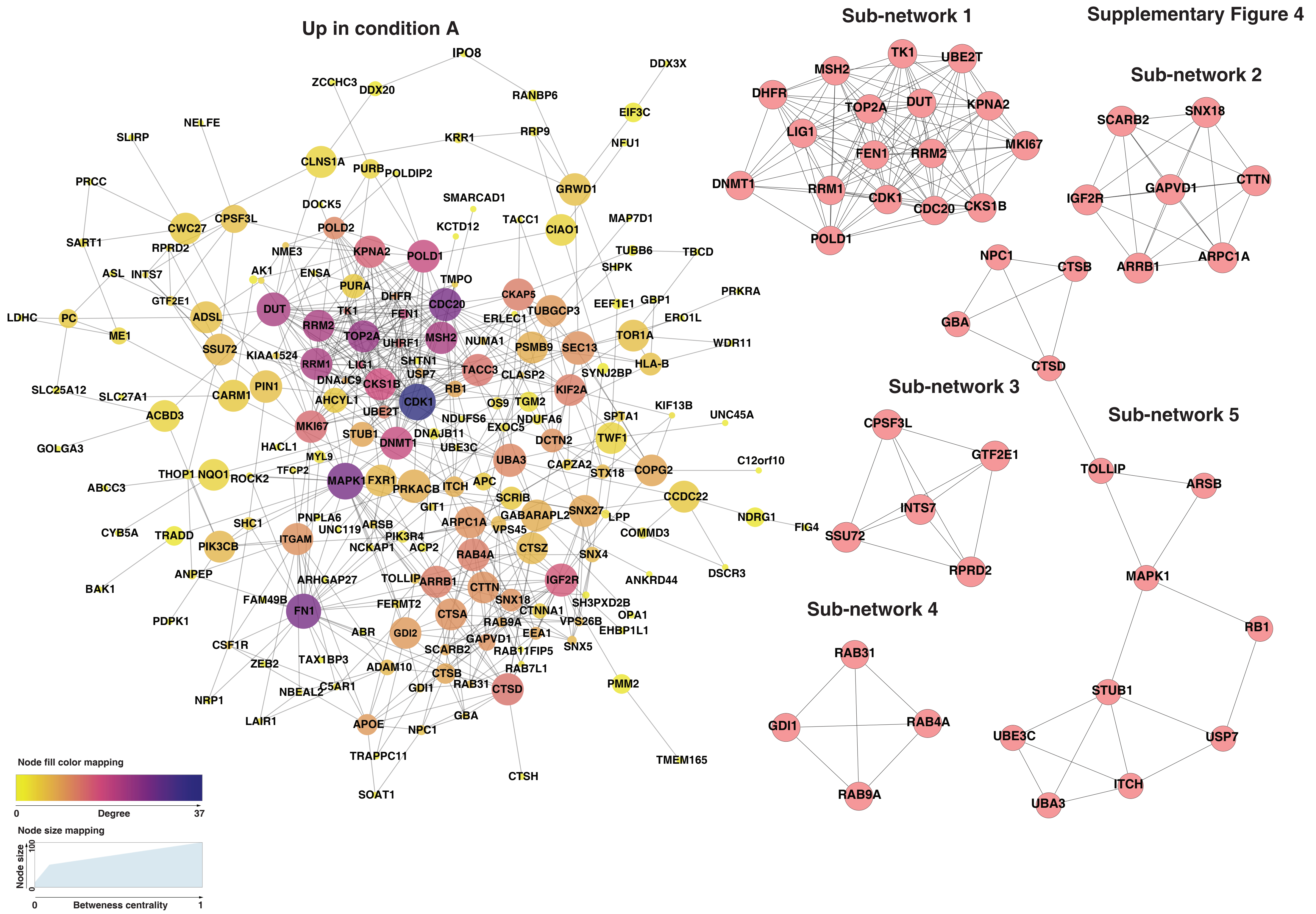

Supplementary Figure 4
Up in condition A
Sub-network 1
Sub-network 2
Sub-network 3
Sub-network 4
Sub-network 5
Node fill color mapping
0
Degree
37
Node size mapping
Node size
0
100
0
Betweness centrality
1

Supplementary Figure 5

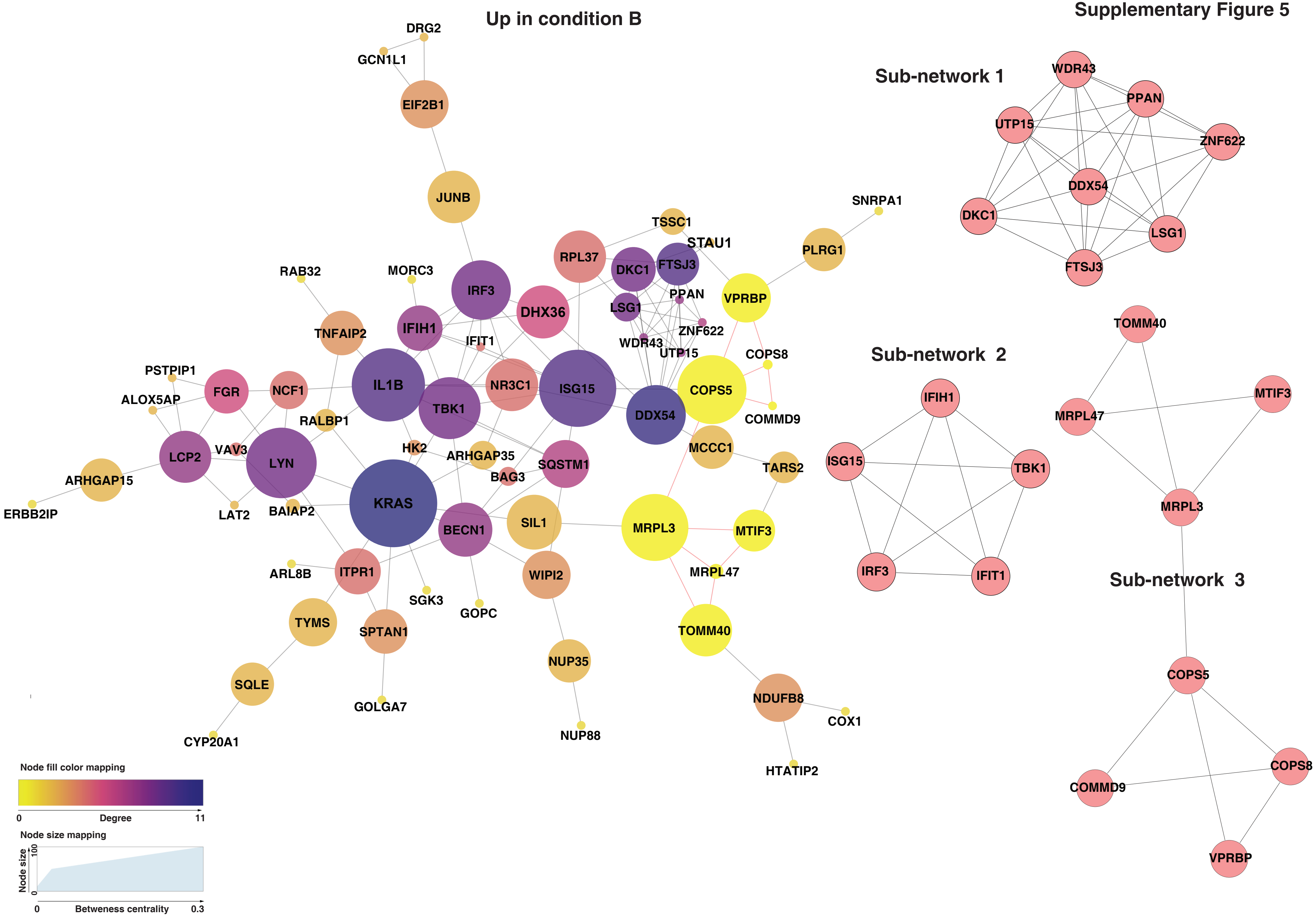

Supplementary Figure 6

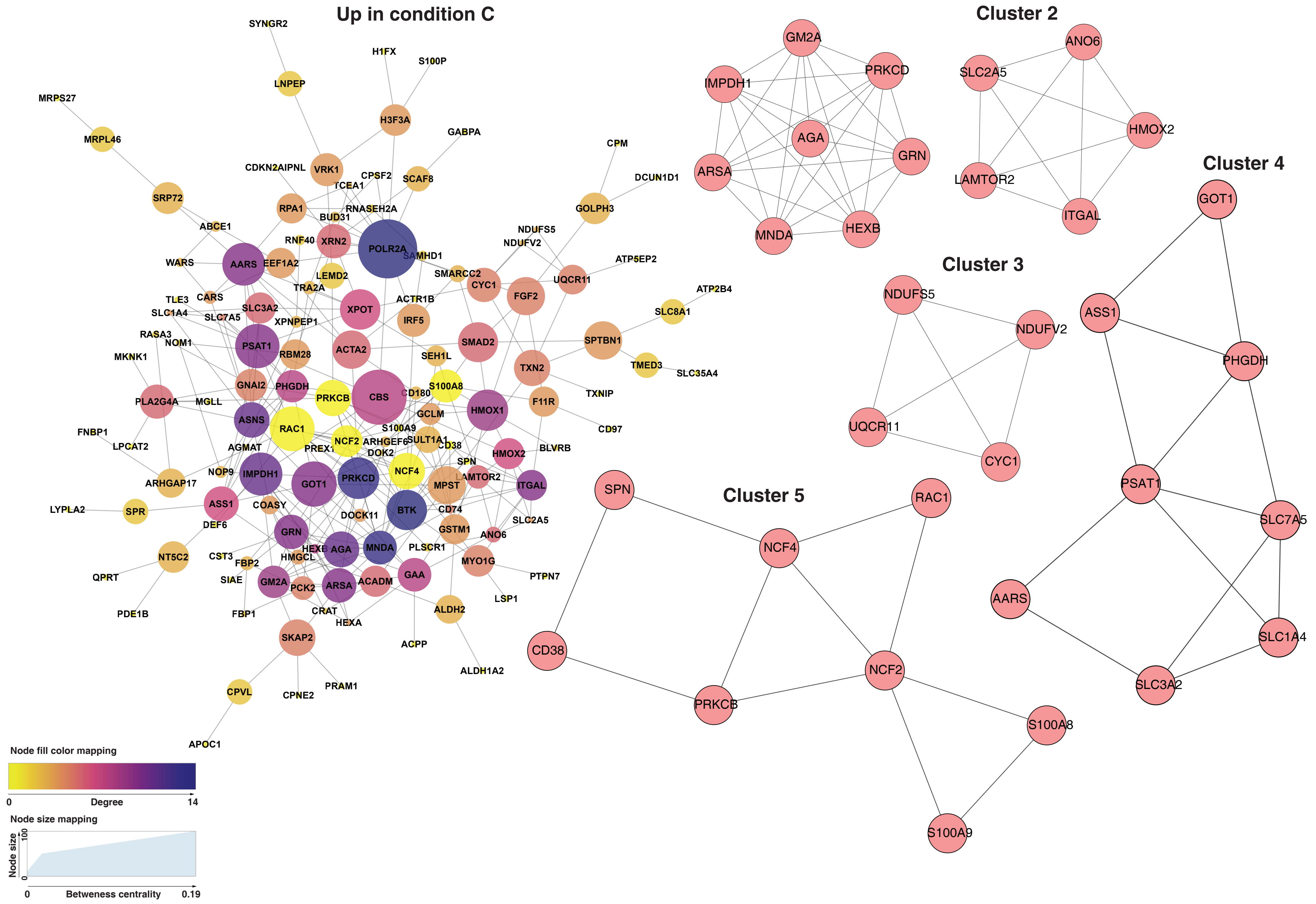

Supplementary Figure 7

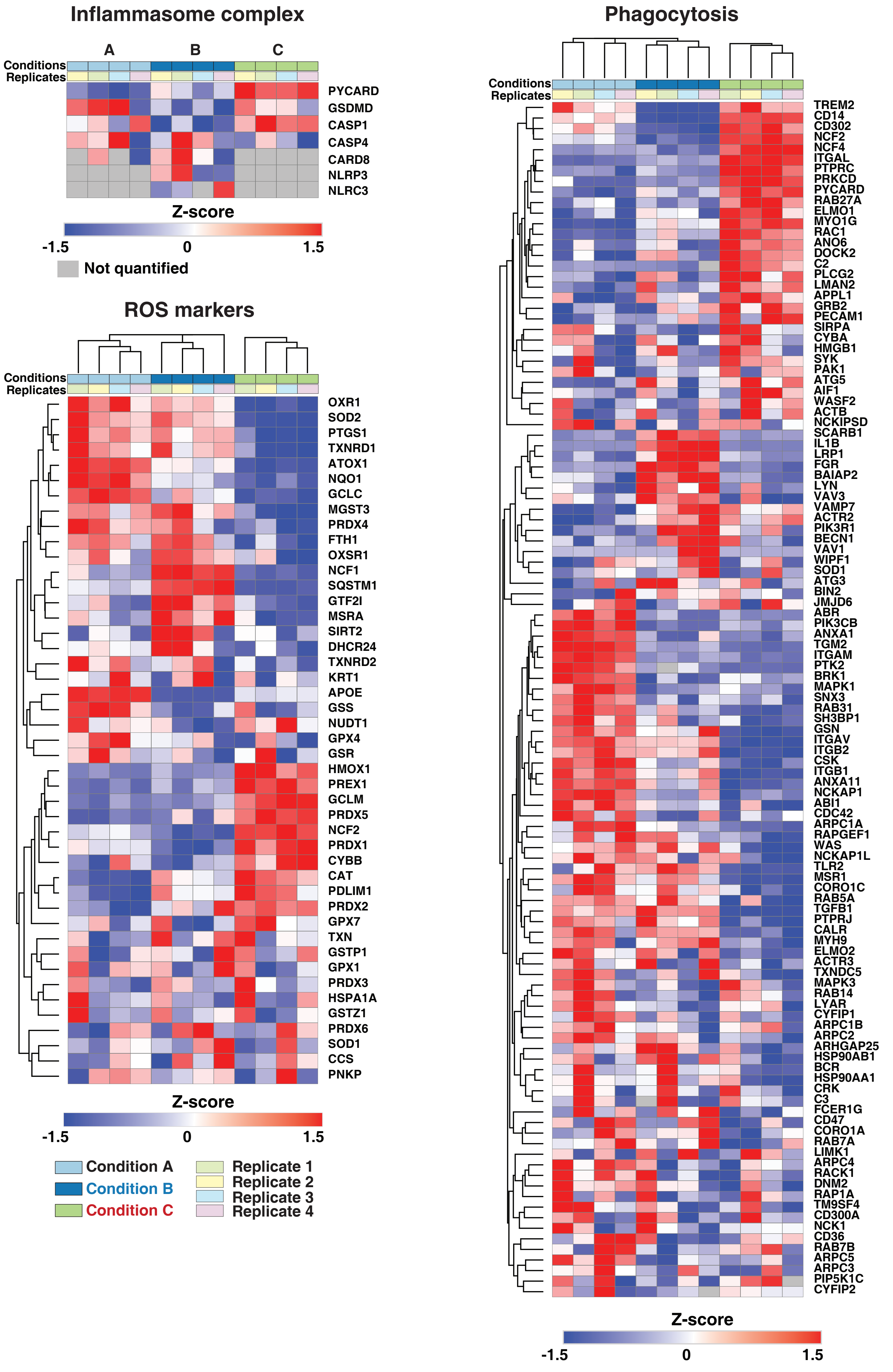

Supplementary Figure 8

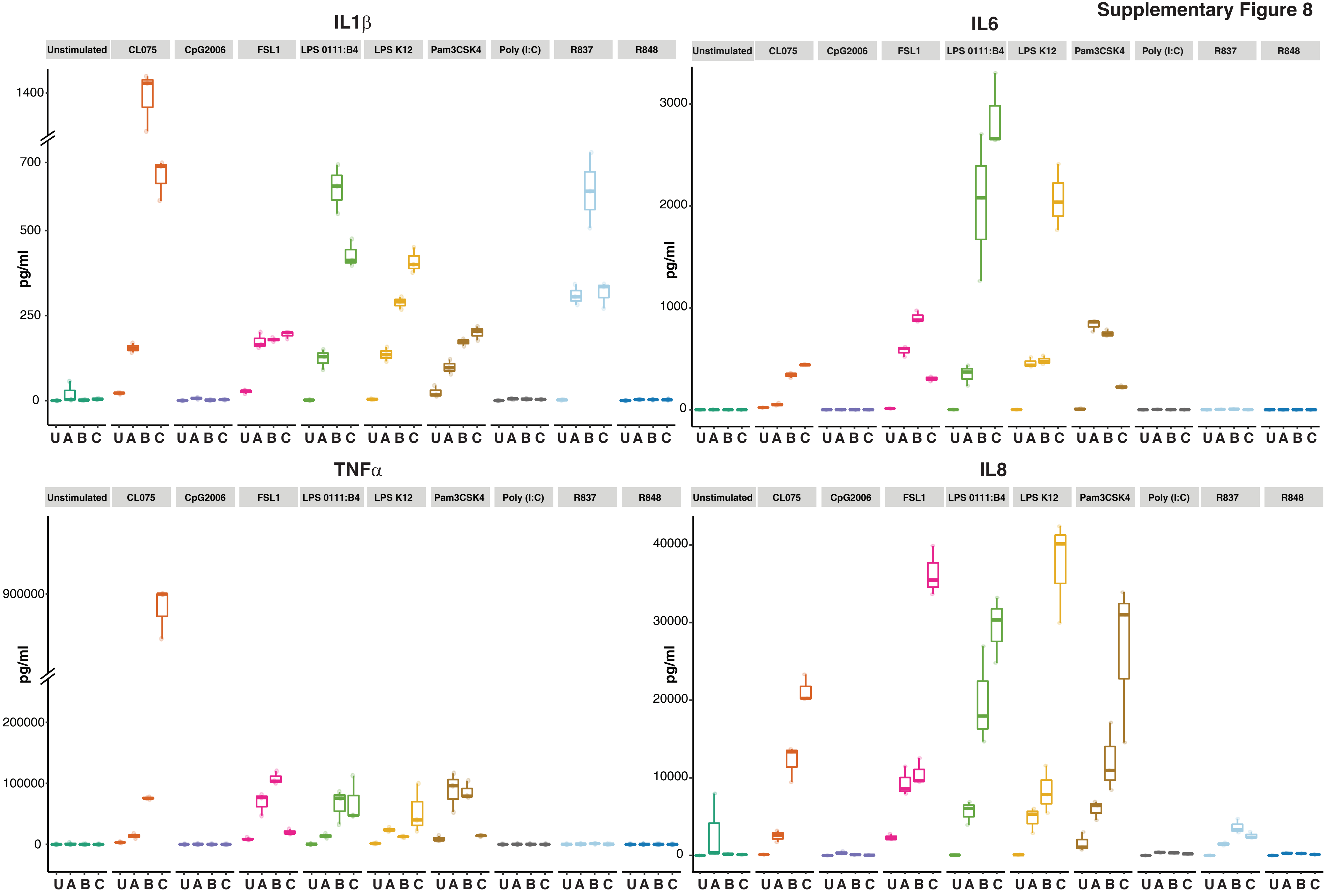

Supplementary Table 1. List of proteins identified from THP-1 macrophages in response to varying cell differentiation protocols using MS/MS

Supplementary Table 2. k-means clustering of identified and quantified proteins from the MS/MS data

Supplementary Table 3. Enriched biological processes in Treatment condition A (Cluster 7) using Enrichr

Supplementary Table 4. Enriched biological processes in Treatment condition B (Cluster 1) using Enrichr

Supplementary Table 5. Enriched biological processes in Treatment condition C (Cluster 9) using Enrichr

Supplementary Table 6. Significantly enriched pathways obtained by Pathway enrichment analysis using Reactome database

Supplementary Table 7. Sub clusters identified using Mcode App (data for Supplementary figures 2-4)

Supplementary Table 8. List of proteins involved in innate immune signaling

Supplementary Table 9. p-values and significance levels for differential cytokine secretion after stimulation with TLR agonists
